# Ancestry Dependent DNA Methylation and Influence of Maternal Nutrition

**DOI:** 10.1371/journal.pone.0118466

**Published:** 2015-03-05

**Authors:** Khyobeni Mozhui, Alicia K. Smith, Frances A. Tylavsky

**Affiliations:** 1 Department of Preventive Medicine, University of Tennessee Health Science Center, Memphis, TN, United States of America; 2 Department of Psychiatry and Behavioral Science, Emory University, Atlanta, GA, United States of America; Yale University, UNITED STATES

## Abstract

There is extensive variation in DNA methylation between individuals and ethnic groups. These differences arise from a combination of genetic and non-genetic influences and potential modifiers include nutritional cues, early life experience, and social and physical environments. Here we compare genome-wide DNA methylation in neonatal cord blood from African American (AA; N = 112) and European American (EA; N = 91) participants of the CANDLE Study (Conditions Affecting Neurocognitive Development and Learning in Early Childhood). Our goal is to determine if there are replicable ancestry-specific methylation patterns that may implicate risk factors for diseases that have differential prevalence between populations. To identify the most robust ancestry-specific CpG sites, we replicate our results in lymphoblastoid cell lines from Yoruba African and CEPH European panels of HapMap. We also evaluate the influence of maternal nutrition—specifically, plasma levels of vitamin D and folate during pregnancy—on methylation in newborns. We define stable ancestry-dependent methylation of genes that include tumor suppressors and cell cycle regulators (e.g., *APC*, *BRCA1*, *MCC*). Overall, there is lower global methylation in African ancestral groups. Plasma levels of 25-hydroxy vitamin D are also considerably lower among AA mothers and about 60% of AA and 40% of EA mothers have concentrations below 20 ng/ml. Using a weighted correlation analysis, we define a network of CpG sites that is jointly modulated by ancestry and maternal vitamin D. Our results show that differences in DNA methylation patterns are remarkably stable and maternal micronutrients can exert an influence on the child epigenome.

## Introduction

Epigenetics refer to a host of molecular mechanisms that can influence phenotypes by regulating gene expression. In humans, much of the research on the epigenome has focused on DNA methylation, partly because methylation is far more amenable to high-throughput and semi-quantitative genome-wide assessments using microarrays. The epigenome-wide surveys have characterized significant inter-individual variability that likely results from a combination of influences that include environmental stimuli [1, 2], diet and medication use [3–6], psychosocial factors [7–9], and physiological changes [10, 11]. While both DNA methylation and gene expression have partially stochastic kinetics [12–14], changes in the methylome may be comparatively stable and serve as reliable indicators of environmental and developmental conditions [15, 16].

There is extensive population and ancestry dependent variation in DNA methylation. According to recent studies, African populations have generally lower global methylation than Caucasians [17, 18]. Additionally, Lam et al. [19] reported that the methylation measured in peripheral blood correlates with leukocyte composition and is associated with ethnicity, psychosocial stress, and early-life socioeconomic status. Studies that have explored genetic regulation of DNA methylation show that variation in the methylome has ancestry-dependent heritability patterns that are modulated by underlying quantitative trait loci or meQTLs [18, 20–24]. The variability in methylation is extensive and it is difficult to disentangle the relative contribution of genetics vs. environment, and to estimate the health implications of individual or ethnic variation.

In the United States, African Americans suffer from disproportionately higher rates of chronic diseases (e.g., diabetes, hypertension, heart diseases) and African American infants also show poorer birth outcomes compared to Caucasian counterparts [5, 25, 26]. The reasons for these persistent health disparities are not entirely clear but are, without doubt, multifactorial and arise from a combination of social, environmental, nutritional, and biological factors. The risk conditions start early in life, as early as the prenatal stage, and epigenetic pathways offer a potential mechanism for perpetuating the effects of early life exposures and setting the stage for future outcomes [25]. Among the many factors, maternal diet and nutritional state are tractable modifiers of the infant epigenome that have impact on offspring health [3, 4, 27, 28]. For instance, folate is a methyl group donor and maternal deficiency is correlated with altered DNA methylation in neonate [29–31]. Similarly, maternal vitamin D is the largest predictor of circulating vitamin D in the neonate, and vitamin D levels are also reported to influence DNA methylation [32–35]. Notably, plasma levels of vitamin D are known to be considerably lower among AAs and this has been attributed to differences in skin pigmentation, availability of vitamin D-binding proteins, and genetic polymorphism [36].

In this study, we systematically compare DNA methylation in neonatal cord blood from African American (AA) and European American (EA) participants of the CANDLE Study (Conditions Affecting Neurocognitive Development and Learning in Early Childhood; http://candlestudy.com) [18, 37–39]. Our goal is to determine if there are replicable ancestry-specific methylation patterns that may implicate risks for diseases that disproportionately affect one group over another. To identify the most robust ancestry-specific CpG sites, we replicate our results in HapMap samples [21]. We then evaluate if maternal micronutrients during pregnancy, specifically maternal vitamin D and folate levels, explain some of the population differences. Finally, we apply a network level analysis to examine if population and nutritional differences influence global patterns in DNA methylation in newborns.

## Materials and Methods

### Study sample

The present study used a subset of the CANDLE samples. Study design and details have been reported [39]. 1,503 healthy women in their second trimester of pregnancy were enrolled between 2005 and 2011 as part of this longitudinal study. Briefly, the inclusion criteria were: a resident of Shelby County Tennessee, able to speak and understand English, age 16–40 years old, and 16–28 weeks of gestation with a singleton pregnancy. All subjects 18 years or older provided written informed consent prior to the assessments. For subjects 16–17.9 years, written informed consent was provided by legally authorized representatives. The study was conducted in accordance with the Helsinki Declaration and was approved and reviewed by the Institutional Review Board of the University of Tennessee Health Science Center. Blood samples were collected from a subset of participants for an ancillary study on molecular biomarkers and 216 cord blood samples were assayed for genome-wide DNA methylation [18, 37, 38, 40]. For this particular report, we limited our analysis to data from self-reported African Americans (N = 112) and European Americans (N = 91) that had DNA methylation data. Samples with self-reported mixed ancestry were excluded.

### Nucleic acid extraction and microarrays processing

Newborn umbilical cord blood was collected at delivery. Whole blood was centrifuged and buffy coat layer isolated and frozen until processed for nucleic acid purification. DNA extraction was performed with Wizard genomic DNA purification reagents (Promega; http://www.promega.com).

Processing of DNA for methylation microarrays is provided in [18, 37, 38]. In brief, 750 ng genomic DNA was bisulfite converted using the EZ DNA methylation kit (Zymo Research; http://www.zymoresearch.com) and interrogated with the Illumina Humanmethylation27 BeadChip (http://www.illumina.com), which assays methylation levels at >27,000 CpG sites. Raw data was processed using the Illumina GenomeStudio (version 2009.1). Level of methylation was estimated by the β value, which is the ratio of fluorescent intensities between the methylated probe and unmethylated probe. This ranges from 0 to 1 and represents the percent methylation at a CpG site. The GenomeStudio software calculates a detection p-value, which estimates the probability that the signal from the target CpG is distinguishable from background noise by comparing the intensity of the target probe against negative control. β values with detection p-value ≥ 0.001 were considered as missing data. Additionally, one probe with a median detection p-value ≥ 10^-6^ across all samples was dropped from analysis. The data was then corrected for batch effects using the COMBAT R package [41, 42]. Following the batch correction, the β values were converted to M-values using a logit transform as described in Du et al. [43]. 5,862 CpG probes that contained a SNP with minor allele frequency greater than 1% in any population, as identified from the 1000 Genomes Project [44], were removed to avoid hybridization artifact (list of these probes in S1 Data). 1,092 probes that target the sex chromosomes were then removed. This resulted in a list of 20,595 quality-checked probes that mapped to annotated genes. The full methylation data is available from the NCBI NIH Gene Expression Omnibus (accession ID GSE64940 at http://www.ncbi.nlm.nih.gov/geo/).

### Plasma vitamin D and folate measurements

Venous blood from the mothers was collected at 16–28 weeks of pregnancy. Blood samples were then centrifuged and serum frozen until processed for micronutrient assays. Serum 25-hydroxy Vitamin D was measured using enzyme immunoassay kit from Immunodiagnostic Systems (IDS; http://www.idsplc.com). This was done at the University of Tennessee Health Science Center. The minimum detection range of the assay is 2 ng/ml. NIST SRM972 Vitamin D was used for quality assurance of 25-hydroxy Vitamin D. The inter-assay variability over the past four years has been less than 6% for the laboratory assay controls. The laboratory participates in the College of American Pathology Quality Assessment Program for monitoring the accuracy and precision of the 25-hydroxy Vitamin D assay and results have been within 1 SD of mean Vitamin D levels [45]. Vitamin D data was available for 147 of the mothers (81 AA, 66 EA) with umbilical cord blood DNA methylation data.

Total folate level in plasma was measured using the 96-well plate adaptation of the *L*. *casei* (ATTC 7469) microbiological assay [46]. This work was performed at the Molecular Epidemiology Laboratory in Birmingham, AL and the method is described in detail in [47]. All measurements were performed within 3 months of sample collection by one research associate throughout the study period using samples that were never subjected to freeze-thaw conditions. Folate data was available from 200 of the mothers (109 AA, 91 EA) with umbilical cord blood DNA methylation data.

### Statistical analysis

Statistical analyses were done on the R platform (http://www.r-project.org) and JMP Statistics (JMP Pro 10.0.0). We applied linear regression to test association between methylation M-values and ancestry (self-reported race). Since maternal age and cellular heterogeneity are known to influence methylation values [17–19], both maternal age and estimated proportions of lymphocytes and granulocytes were used as covariates in the regression model. Birth weight only has limited influence on DNA methylation and this was not added as a factor in the regression model [38]. For association with maternal nutritional factors, the M-values were regressed on maternal plasma vitamin D or folate with race, maternal age, and estimated blood cell counts as covariates. P-values were adjusted for false discovery using the Benjamini and Hochberg method [48]. Enrichment in cis-meQTLS among CpG sites with population difference was evaluated using the hypergeometric test. Gene ontology and pathway enrichment analysis was done using DAVID 6.7 [49] (http://david.abcc.ncifcrf.gov).

### Replication in HapMap data

The HapMap data we used was provided by Fraser et al [21]. It compares between 30 CEU and 30 YRI trios. We obtained the full list of uncorrected p-values (based on Wilcoxon tests) and used this to evaluate how many of the differentially methylated sites we identified in CANDLE at FDR 5% are also differentially methylated in the HapMap panel using these criteria: (1) uncorrected p-value ≤ 0.05 between YRI and CEU, and (2) consistency in either higher or lower methylation in African ancestry in both the CANDLE and HapMap groups.

### Estimation of blood cell counts

Data from leukocyte subtypes (GEO GSE35069) was used to identify cell type specific CpG sites, and the method described by Houseman and colleagues was used to estimate the proportion of granulocytes and lymphocytes in our whole blood DNA samples [50, 51].

### Network analysis

We used the WGCNA R package to define correlated networks in the CANDLE cord blood methylome [52, 53]. This is a dimension reduction procedure originally developed for transcriptomic data and the computational details are described in Zhang and Horvath [54]. This method has been adapted to analyze co-methylation networks [22, 55, 56].

WGCNA is based on the pair-wise variance and correlation structure among genes. We used the set of 20,595 probes for network construction and applied standard parameters described in [54] (detail on network construction in S1 Text). WGCNA generates a gene-by-gene similarity matrix (20,595 x 20,595 matrix) based on pair-wise Pearson correlations between nodes (i.e., probes targeting methylation sites). In the second step, the similarity matrix is transformed into an adjacency matrix that has a scale-free network topology using a soft thresholding power function, β, that is chosen to fit a scale-free network using linear regression model fitting index, R^2^ (β = 6, R^2^ = 0.854, mean connectivity or mean k = 25, max k = 295). Third, the topological overlap matrix (TOM) is defined to estimate network connectivity between nodes. Then networks of tightly inter-correlated transcripts or modules are defined by hierarchical clustering. We have labeled the modules as Meth1 to Meth9 based on module size (i.e., from largest to smallest depending on the number of probe members). All probes that do not fit into any module are placed in a separate bin (here represented by Meth0).

After defining the modules, WGCNA provides intra-modular network connectivity values for each gene to help identify hub genes. Furthermore, the module eigengene or ME (first principal component) provides a single vector that represents the summarized variation of a co-methylation network and can be used to examine inter-module relatedness and association with other factors. To test relationship between the module eigengenes and the different population variables (Table 1), we first applied simple bivariate analysis. For ME associated with race and vitamin D, we then applied multiple linear regression analysis with race, vitamin D, and race x vitamin D interaction as predictors.

**Table 1:** Participant characteristics.

Variables	African AmericansN or Mean (SD) ^1^	European AmericansN or Mean (SD) ^1^	p-value^2^
N	112	91	
Child sex	53 females	50 females	ns
	59 males	41 males	
Gestational age (weeks)	38.84 (1.51)	39.15 (0.91)	ns
Maternal age (years)	25.75 (5.03)	29.45 (4.50)	<.0001
Birth weight (Kg)	3.18 (0.47)	3.52 (0.42)	<.0001
Plasma Vitamin D (ng/ml)	17.74 (6.03)	20.68 (6.23)	0.004
Plasma folate (ng/ml)	28.30 (18.40)	32.84 (12.16)	0.05
Estimated lymphocyte fraction	38.08 (12.11)	42.73 (10.23)	0.004
Estimated granulocyte fraction	48.72 (13.01)	45.84 (10.51)	ns
Average of all CpG sites (M-value)	-2.69 (0.15)	-2.63 (0.15)	0.01

^1^ Child race based on maternal report of parents’ race

^2^ Chi-square test (for child sex) and analysis of variance (for continuous variables) with race as predictor

## Results

### Analysis of DNA methylation in CANDLE

We used methylation microarray data from cord blood of 112 AA and 91 EA newborns (previously reported in Adkins et al. [18]). Table 1 shows maternal and child characteristics and variables that are significantly different between AAs and EAs (i.e., maternal age, birth weight, plasma vitamin D, folate, and estimated lymphocyte fraction). The methylome data we analyzed consists of 20,595 probes that target 15,280 promoter CGIs and 5,315 non-CGIs outside of promoter regions. We applied linear regression to evaluate variation in methylation M-values as a function of population group (AA or EA). Since maternal age and blood cell counts have significant influence on DNA methylation [19, 37] and both show significant difference between AA and EA in CANDLE (Table 1), these were included as covariates. At an FDR corrected p-value ≤ 0.05, methylation at 3,802 sites showed significant difference between AAs and EAs (S2 Data). This is over 18% of the methylome surveyed. Of these, 70% (2,647 CpGs) have lower methylation in AAs and only 30% (1,155 CpGs) have higher methylation in AAs (Table 2). This is consistent with previous observation that AAs exhibit lower overall methylation [17, 18]. The average methylation of all CpG sites is also significantly lower in AAs (p-value = 0.01; Table 1). None of the other variables listed in Table 1 are significant predictors of average methylation.

**Table 2:** Number of CpGs that are differentially methylated.

		AA vs EA in CANDLE		Ancestry-dependentCANDLE and HapMap	
	Total	Low AA	High AA	Low AA/YRI	High AA/YRI
**CpG counts**	20,595	2,647	1,155	1,055	319
**Cis-meQTLS counts**	333	85	74	32	48

### Identifying stable and consistent ancestry-dependent DNA methylation

The divergence between AA and EA in methylation may be due to a combination of environmental and genetic factors. To estimate the extent of ancestry-dependent divergence, we examined what fraction of CpG sites that are differentially methylated between CANDLE AA vs. EA populations are also differentially methylated between YRI (Yoruba in Ibadan, Nigeria) vs. CEU (Utah European ancestry from CEPH panel) in HapMap. While the CANDLE data is from cord blood, the HapMap data is from transformed cell lines, and only robust and stable effects will be detected. Out of the 3,802 differentially methylated sites identified in CANDLE, 1,374 also show consistent differential methylation in HapMap (at lenient nominal p-value < 0.05 and with the same direction of association, i.e., either higher or lower methylation M-values in the African ancestral groups, AA and YRI, relative to European ancestral groups, EA and CEU; S3 Data). We consider these as ancestry-specific methylation sites. Of these, 1,055 CpGs (77%) have lower methylation in AA/YRI and 319 CpGs (23%) have higher methylation in AA/YRI (Table 2). This shows that more than 36% of the differentially methylated sites and the overall lower methylation in African groups are replicable across cell types and age. A number of CpGs are in genes implicated in familial colorectal cancer and tumor suppression, e.g., neuroblastoma RAS viral (v-ras) oncogene homolog (*NRAS*; cg07068998), adenomatosis polyposis coli (*APC*; cg24332422), mutated in colorectal cancers (*MCC*; cg06894812), breast cancer 1, early onset (*BRCA1*; cg19531713) (see S3 Data for enriched gene sets). Other genes with ancestry-specific methylation include the Duffy blood group atypical chemokine receptor gene (*DARC*; cg18552413), which has a null mutation in Africans and attributed with blood phenotypes [57, 58], and two genes involved in DNA methylation and repair: DNA (cytosine-5-)-methyltransferase 1 (*DNMT1*; cg17445987) and bromodomain adjacent to zinc finger domain 2A (*BAZ2A*; cg14634319).

### Genetic regulation of ancestry-dependent DNA methylation

We examined if the differentially methylated sites in CANDLE are associated with *cis*-acting genetic regulation. A comprehensive meQTL analysis has been done for this data [24] and using meQTL information from this previous work, we counted the number of CpGs that are modulated my *cis*-meQTLs. For the 3,802 sites with methylation difference between AAs and EAs, 159 are associated with at least one nearby meQTL marker (Table 2). This is over 2.5-fold enrichment in *cis*-meQTLs among the genes that are differentially methylated (hypergeometric p-value of 2.9 × 10^-35^) and indicates that some of the population variation is due to genetic variation. However, *cis*-meQTLs alone are unlikely to explain the hypomethylated state in African ancestral group and only 32 of the replicated CpGs with low methylation in AA/YRIs are modulated by *cis*-meQTLs in CANDLE (Table 2).

### Effect of maternal vitamin D and folate on newborn methylation

We next evaluated if nutritional differences could contribute to the population variation. Specifically, we examined association between maternal plasma levels of folate and vitamin D measured during mid-pregnancy (16–28 weeks) and DNA methylation in newborns. Plasma levels of folate ranged widely from 4.84 to 109.14 ng/ml. While none of the mothers were deficient, AA mothers had modestly lower folate levels than EA mothers (p-value = 0.05; Table 1). Plasma 25-hydroxy vitamin D ranged from 9.4 to 35.2 ng/ml, and 51 AA mothers and 28 EA mothers showed levels below the recommended concentration of 20.0 ng/ml [59, 60]. Consistent with other studies [35, 36, 61, 62], plasma vitamin D was significantly lower in AAs compared to EAs (p-value = 0.004; Table 1).

To test association between maternal micronutrients and newborn methylation, we first performed simple linear regression. Both vitamin D and folate levels showed only nominally significant effects. Vitamin D had the most significant association with the methylation of transducin-like enhancer of split 1 gene (*TLE1*; cg15915418; unadjusted p-value = 0.00006) and folate had the most significant association with methylation of WD repeat domain 5 (*WDR5*; cg03243700; unadjusted p-value = 0.0002). None of the CpG sites passed the 5% FDR threshold (all nominal p-values and statistics are provided in S2 Data). Regressing methylation levels on vitamin D or folate with race, maternal age, and estimated blood cell counts as covariate resulted in no significant association at 5% FDR (S2 Data).

### Global organization of co-methylation networks in CANDLE cord blood

We next applied weighted gene co-expression network analysis (WGCNA) to evaluate if maternal factors influence the global network organization of the methylome. We applied WGCNA to the set of 20,595 CpGs and this organized the methylome into 9 modules ranging in size from 7,924 to 160 network members, labeled as Meth1 to Meth9 (Table 3; S4 Data). Each module represents a network of CpG sites that have highly correlated variation in methylation across the CANDLE samples. Methylation of 709 CpGs showed low connectivity and did not fit into any module (relegated to module Meth0). Using gene ontology (GO) enrichment we found six modules with significant functional enrichment at FDR ≤ 0.05 (Table 3; module characteristics and GO profiles are provided in S4 Data). These modules are networks of genes related to immune response (Meth2, Meth4, Meth5), regulation of cell cycle and cell death (Meth1 and Meth8), and neuron differentiation (Meth3).

**Table 3:** Co-methylation modules defined in CANDLE newborn cord blood.

		Gene Ontology (GO) enrichment^1^						
Module	Size	Top GO	p-value	FDR	Ancestry^2^	Vit D^2^	Lymph^2^	Gran^2^
**Meth1** ^**3**^	7924	GO:0010605~negative regulation of macromolecule metabolic process	3.1E-11	1.6E-07	0.03			
		GO:0042981~regulation of apoptosis	3.3E-11	8.4E-08				
**Meth2**	2430	GO:0006952~defense response	6.5E-14	2.6E-10				
**Meth3**	2231	GO:0030182~neuron differentiation	4.4E-11	1.8E-07				
**Meth4**	2104	GO:0006952~defense response	3.9E-08	1.35E-04	0.02	0.007		
**Meth5**	1917	GO:0006955~immune response	1.4E-18	5.5E-15	4.3E-05	1.8E-61	9.8E-50	
**Meth6**	1895	GO:0010522~regulation of calcium ion transport into cytosol	2.0E-05	0.07	0.02	0.02	0.007	
**Meth7**	641	GO:0010033~response to organic substance	2.8E-05	0.07	0.0004	0.006		
**Meth8**	584	GO:0043067~regulation of programmed cell death	6.4E-07	0.002				
**Meth9**	160	GO:0051252~regulation of RNA metabolic process	0.002	0.8	0.03			

^1^Top Biological Process GO term; enrichment p-value based on modified Fisher’s exact test from DAVID and Benjamini FDR corrected p-value (http://david.abcc.ncifcrf.gov).

^2^Association between module eigengenes and ancestry, maternal vitamin D, and estimated fraction of lymphocytes (Lymph) and granulocytes (Gran) based on simple regression analysis; these are uncorrected p-values.

^3^Meth1 is the largest module and the GO analysis was done for the top 5,000. Top 2 GO categories shown.

**Fig 1:**
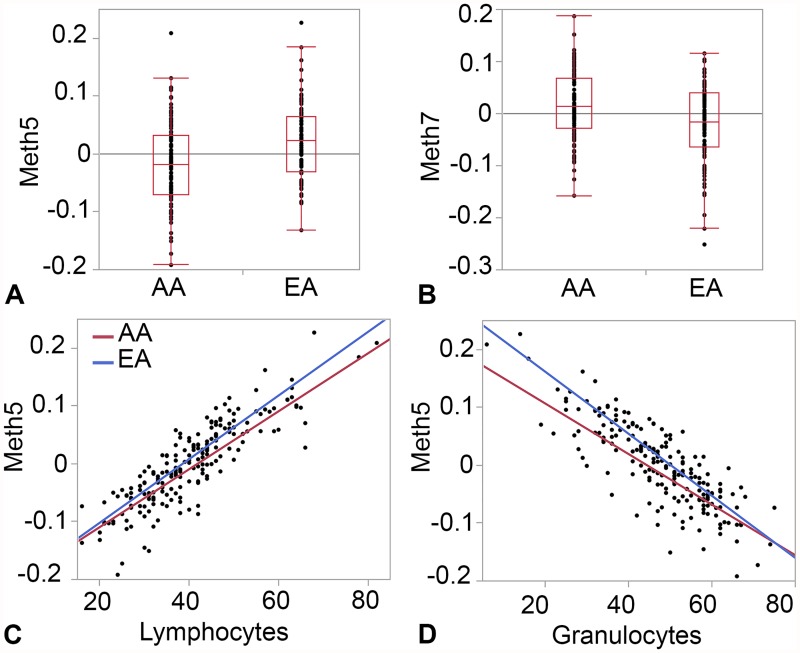
Ancestry-specific methylation modules in CANDLE. Box plots show differences in module eigengenes between African Americans (AA) and European Americans (EA) for Meth5 (**A**) and Meth7 (**B**). Scatter plots show the correlation between the cell-type specific Meth5 module and estimated fractions of lymphocytes (**C**) and granulocytes (**D**).

For each co-methylation module, the collective variance captured by the correlated network of CpGs can be summarized by a single eigenvector (first principal component), also known as the module eigengene or ME (ME values provided in S4 Data). This reduces the high-dimensional data to just 9 MEs that can then be related to other factors that may contribute to the covariance structure. Using the MEs, we applied simple linear regression to test if any of the variables in Table 1 are associated with the co-methylation networks. Race has the most extensive influence and is a significant predictor for 5 of the 9 modules at nominal p-value ≤ 0.05 (Table 3). Of these, Meth5 and Meth7 pass the Bonferroni corrected p-value ≤ 0.05 threshold (unadjusted p-value ≤ 0.006 for 9 tests) (Fig. 1A, 1B). The strongest effect of race is on the immune module, Meth5, a network with 1,917 CpG members. We note that Meth5 is also a cell type specific network and is strongly correlated with estimated proportions of lymphocytes (R = 0.86 in AAs and R = 0.87 in EAs; p-value < 0.0001) and granulocytes (R = -0.80 in AAs and R = -0.87 in EAs; p-value < 0.0001) (Fig. 1C, 1D). Our data indicate difference in estimated lymphocytes fraction between AAs and EAs (Table 1) and since such variation in composition of blood cells can contribute to variation in methylation, we performed multiple regression to evaluate if the association of Meth5 with race can be accounted by differences in lymphocyte and granulocyte counts. This showed that both self-reported race and cell estimates are significant predictors of Meth5 and collectively contribute to the variation in methylation of the CpGs members (S4 Data). As expected, the top hub genes (the genes with highest intra-modular connectivity within the respective module) in the ancestry-specific modules are those with significant differential methylation between AAs and EAs. For example, top hub genes in Meth5 have lower methylation in AAs and top hub genes in Meth7 have higher methylation in EAs (Fig. 1; see S2 Data for gene-level intra-modular connectivity and module membership).

**Fig 2:**
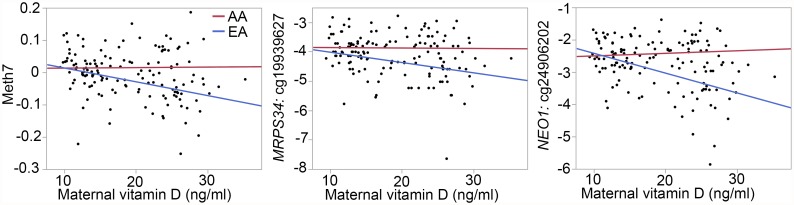
Interaction between ancestry and vitamin D in the Meth7 co-methylation network. Meth7 is a co-methylation network that is influenced by both ancestry (based on self-reported race by mothers) and levels of maternal vitamin D. These are regression plots for the Meth5 module eigengene, and the top two hub genes in Meth5: *MRPS34* and *NEO1*. Average methylation of the CpG network is higher in African Americans (AAs). Negative correlation between vitamin D (ng/ml) and methylation levels (M-value) is seen only in European Americans (EAs; for EAs, R = -0.35, p-value = 0.004 for Meth7 and vitamin D; R = -0.28, p-value = 0.025 for *MRPS34* and vitamin D; and R = -0.46, p-value < 0.0001 for *NEO1* and vitamin D).

Meth7 is a network of 641 CpG sites and enriched in genes involved in response to organic substance (GO:0010033; Table 3). In addition to ancestry, maternal vitamin D is another significant predictor of Meth7 (Table 3). To explore potential interaction between ancestry and vitamin D on Meth7, we used the ME as response variables and applied multiple linear regression with race, vitamin D, and race x vitamin D interaction as predictors. This showed that Meth5 is influenced by both race and maternal vitamin D with significant race x vitamin D interaction (Table 4). The hub CpGs have significantly higher methylation among AAs (at FDR 5% criterion) and are also associated with maternal vitamin D. The regression plots for Meth7 and the constituent CpGs show that average methylation is higher in AAs, and methylation is negatively correlated with vitamin D but this effect is seen mainly in EAs (Fig. 2; plots for only the top two hub CpGs are shown). Taken together, Meth7 represents a correlated network of CpGs that is jointly modulated by ancestry and vitamin D.

**Table 4:** Summary of multiple regression model for Meth7.

	Meth7			
Predictor variables^1^	Coeff	SE	t-Ratio	p-value
Race	0.02	0.006	3.36	0.001
Vitamin D	-0.002	0.0009	-2.19	0.03
Race x Vitamin D	0.002	0.0009	2.36	0.02

^1^ Result for the full model with predictors race, vitamin D, race x vitamin D is R^2^ = 0.16, F_(3, 139)_ = 8.74, p-value < 0.0001 for Meth7

Unlike maternal vitamin D, maternal folate is not associated with any ME. Other variables in Table 1 with significant association with the MEs include birth weight with Meth6, and child sex with Meth9 (full bivariate statistics of predictors and MEs are provided in S4 Data).

## Discussion

We have described population differences in methylation patterns that can be consistently detected and are robust ancestry markers in two different study cohorts. Particularly intriguing is the overall lower methylation in the African groups in both neonatal cord blood that compared between AAs and EAs, and in transformed cell lines from the HapMap panel that compared between Yoruba Africans and CEPH Europeans. We considered nutritional differences, particular plasma 25-hydroxy vitamin D, which is known to be generally lower among people of African ancestry, as a factor that may contribute to this population variation. Using a weighted network approach, we define a module of CpG sites (a co-methylation network) whose correlated variance in the CANDLE population is associated with race, maternal vitamin D levels, and an interaction between race and vitamin D.

### Multiple modulators influence DNA methylation

The methylome is shaped by multiple factors. Parsing the relative contribution of these variables, which include genetic, environmental, lifestyle as well as interactional effects (gene x gene, gene x environment) is challenging. Recent studies have shown that maternal nutrition during pregnancy, psychosocial stress, and even socioeconomic status (SES) at an earlier time point can contribute to population variation in DNA methylation [3, 19]. In our present study, we did not evaluate the effect of SES or maternal education and stress. Another limitation is that we relied solely on self-reported race from mothers and this fails to take into account admixture effects. However, replication of these findings in an independent cohort of African and European ancestral samples in HapMap supports the robustness of our results and suggests that misclassification is not likely to substantially influence the results. We would have preferred to further test the consistency of our findings in another cord blood data to discern the effects that are specific to newborns. However, we found no other cord blood methylation data from a similar cohort with equal racial diversity. Presumably, the environmental context and social conditions differentiating the AAs and EAs in the Memphis area are different from that in the very disparate HapMap populations, i.e., YRIs (Yoruba Nigerians) and CEUs (Utah residents with European ancestry). The global hypomethylation in the two African ancestral groups may therefore be due to factors that are more proximate to the DNA methylation pathways and generalizable across different social and environmental settings. Nonetheless, SES and the many variables that correlate with social inequality can have a pervasive influence on an individual’s diet, stress physiology, and general health. A more comprehensive study that includes macro- and micronutrients, metabolic markers, SES, and psychosocial stress remains to be done.

Here we note that both the CANDLE and HapMap methylome were quantified using Illumina 27K arrays and technical artifacts can be a potential source for some of the population effects. For instance, one could speculate probe design artifacts that bias more efficient hybridization for one ancestral group compared to another. However, a study that measured methylation in leukocytes using [^3^H]-methyl acceptance assay [17] also found a generally hypomethylated state in AAs compared to EAs. The hypomethylation we have observed in the current data is therefore unlikely to be an array specific effect. Nevertheless, we must emphasize that the 27K array by no means provides a comprehensive coverage of the methylome [63]. The ~27,000 probes interrogates only a fraction of CpGs and none of the non-CpG methylation sites [64]. Within the confines of this limited view of the human methylome, our results demonstrate an overall lower methylation in African ancestral groups that is robust and independent of cell type and age.

### Genetic regulation of DNA methylation

Genetic variation most certainly contributes to some of the differences in methylation profiles. Recent studies have defined significant heritability and genetic regulation in DNA methylation in different cell types [18, 20–24, 65]. While we see enrichment in *cis*-meQTLs among the genes that show differential methylation, it is unlikely that *cis*-effects alone explain the global hypomethylation in the AAs and YRIs since we expect individual alleles with positive additive effect on methylation to average out between both European and African ancestral groups. In other words, multiple *cis*-acting variants distributed across the genome that lower methylation of CpGs should not show higher allele frequency in the African populations simply by chance.

Alternatively, the global downregulation in AAs and YRIs could be due to one or more *trans*-acting variants that have widespread effect on the epigenome. In such a scenario, pertinent candidates would include regulators of methylation and demethylation pathways such as the DNA (cytosine-5-)-methyltransferase (*DNMT*) and tet methylcytosine dioxygenase (*TET*) genes [66]. We did not examine *trans*-acting meQTLs in this present study but other groups have used comparable sample sizes for candidate gene and genome-wide exploration of *trans*-meQTLs [22, 67]. An example is the candidate gene analysis by Bell et al. [67] that found multiple *trans*-meQTLs map to SNPs near *DNMT1*. Variants in this gene and other DNA methylation regulators are viable candidates that could have a global effect on the methylome.

### Nutritional difference and effect on gene methylation

Studies in both animal models and humans show that the epigenetic state is particularly sensitive to nutritional factors and maternal dietary differences can have far reaching effects on child development [3, 27, 68, 69]. We propose that nutritional differences, particularly of nutrients that are known to be variable between African and European groups, could result in the distinct methylation profiles. Vitamin D is one such factor that has lower plasma levels in people of African descent compared to Caucasians [35, 36, 61, 62]. In fact, currently there is widespread global vitamin D deficiency including in parts of Africa and Asia, which may pose a major health challenge [70]. There is also an emerging role for vitamin D in the prevention of chronic diseases including cancer, diabetes, and dementia [70–72] and we consider this an important micronutrient to evaluate in the context of health disparities and epigenetics. Other nutrients that can influence gene methylation are those that are involved in single carbon metabolism (e.g., folate, B vitamins, methionine) [73, 74].

In the present study, we considered the influence of only two micronutrients measured at 16–28 weeks of pregnancy in the CANDLE mothers. We found no deficiency but modestly lower folate levels in AA compared to EA mothers. 25-hydroxy Vitamin D, on the other hand, had high prevalence of deficiency according to the recommended plasma concentration of ≥20.0 ng/ml [59, 60] and was significantly lower among AAs [35, 36, 61, 62]. As folate is directly involved in methyl-group metabolism, there have been more studies examining the relation between folate and the epigenome. Some studies, particularly in animal models, have found significant influence of maternal folate [29–31], but others have found no association between maternal folate and DNA methylation in offspring [3, 73, 74]. There have also been studies on the relation between vitamin D and DNA methylation and these are starting to reveal a role for vitamin D in shaping the epigenome [32, 34, 35].

The gene-level analysis we performed found only limited influence of these nutrients on DNA methylation. Since this is on a genome-wide scale, we reason that the nutrients may exert only a small effect on any one site and the likelihood of detecting an individual effect is low after multiple test correction. To more effectively capture the summation effect of multiple sites, we applied a weighted correlation method [22, 55, 56]. This essentially reduces the data to fewer dimensions, and aggregates genes with shared variance into tightly correlated networks. We find that race and maternal vitamin D are influential factors at the network level. The ancestry-specific module, Meth7, enriched in genes involved in response to organic substance, represents a co-methylation network that is jointly modulated by race and vitamin D with significant interaction between the two predictors.

Of the 641 CpG members in Meth7, 217 have significant variation between AAs and EAs (at FDR 5%) and 240 are associated with maternal levels of vitamin D (at nominal p-value of 0.05). However, we should note that unlike the overall trend, 214 of the 217 differentially methylated CpGs in this module have higher methylation in AAs. The ME of this module is negatively correlated with vitamin D but this effect is seen mainly in the EA subset. This indicates that higher vitamin D among EAs is associated with lower methylation of CpGs that belong to this module. This shows a more complicated relationship between ancestry, vitamin D, and DNA methylation. The influence of vitamin D on Meth7 does not explain the global hypomethylation among AAs relative to EAs. What our results show is simply that circulating levels of vitamin D and ancestry both exert influence on the methylome, and the effect of maternal vitamin D on neonate methylome appears stronger than that of folate.

### Health implications

The genes that show hypomethylation in the AA/YRI groups are enriched in tumor suppressors and cell cycle regulators (S3 Data). The four genes linked to colorectal cancer (*NRAS*, *PIK3CA*, *MCC*, *APC*) all show lower methylation in the AA/YRI groups. This observation is particularly important in light of the documented racial disparity in the occurrence of certain cancers and chronic diseases and differences in disease progression and mortality [25, 75, 76]. Colorectal cancer has a much higher prevalence among AAs and this is associated with higher expression of genes involved in cell cycle regulation [77, 78]. However, there is also evidence that differences in cancer prevalence arise from socioeconomic and cultural variables [79, 80]. The intersection between the various biological, social, and environmental factors could very well leave a mark in the epigenome, and these basal differences in newborn methylation could be a predisposing factor for later disease development.

### Conclusion

In summary, our results show that ancestry and maternal circulating levels of vitamin D have a joint influence on DNA methylation in infants but vitamin D differences do not explain the lower overall methylation in African ancestral groups. A number of other nutrients and social and environmental variables will have to be factored in to draw a more comprehensive picture. The profile of genes that show ancestry-dependent methylation (e.g., *MCC*, *APC*, *BRCA1*) and the potential role of maternal nutrition in shaping the methylome of newborns have important health implications.

## Supporting Information

S1 Data List of filtered probes with SNPs. List of probes that overlap SNPs defined in the 1000 Genomes Project.
(XLSX)

S2 Data Regression analysis and module membership results for the full data. Regression statistics (methylation as a function of ancestry, vitamin D and folate) and network membership results for the full list of CpG sites.
(XLSX)

S3 Data Ancestry-specific CpG sites and enriched gene functions. This provides the list of ancestry-specific CpGs and enriched gene functions.
(XLSX)

S4 Data WGCNA module characteristics and regression results. This provides module eigengene values, module characteristics, and association with maternal and child variables.
(XLSX)

S1 Text Method on weighted correlation network construction. This provides more detail on how the co-methylation modules were constructed.
(PDF)
