# Supplementary material for: Ancestry Dependent DNA Methylation and Influence of Maternal Nutrition
Source: PLoS One. 2015 Mar 5;10(3):e0118466. doi: 10.1371/journal.pone.0118466 (PMC4350920; doi:10.1371/journal.pone.0118466)
Supplement: S1 Text — This provides more detail on how the co-methylation modules were constructed. (PDF) [file pone.0118466.s005.pdf]

Text S1

Weighted correlation network construction

We used the WGCNA R package for network construction. The methylation data was loaded as a 203 x 20,595 matrix (203 samples and 20,595 probes). Hierarchical clustering was then done using the flashClust function and the sample tree was cut to remove four samples that appeared to be outliers (**Figure 1**). The data from the 199 samples were used for co-methylation network analysis.

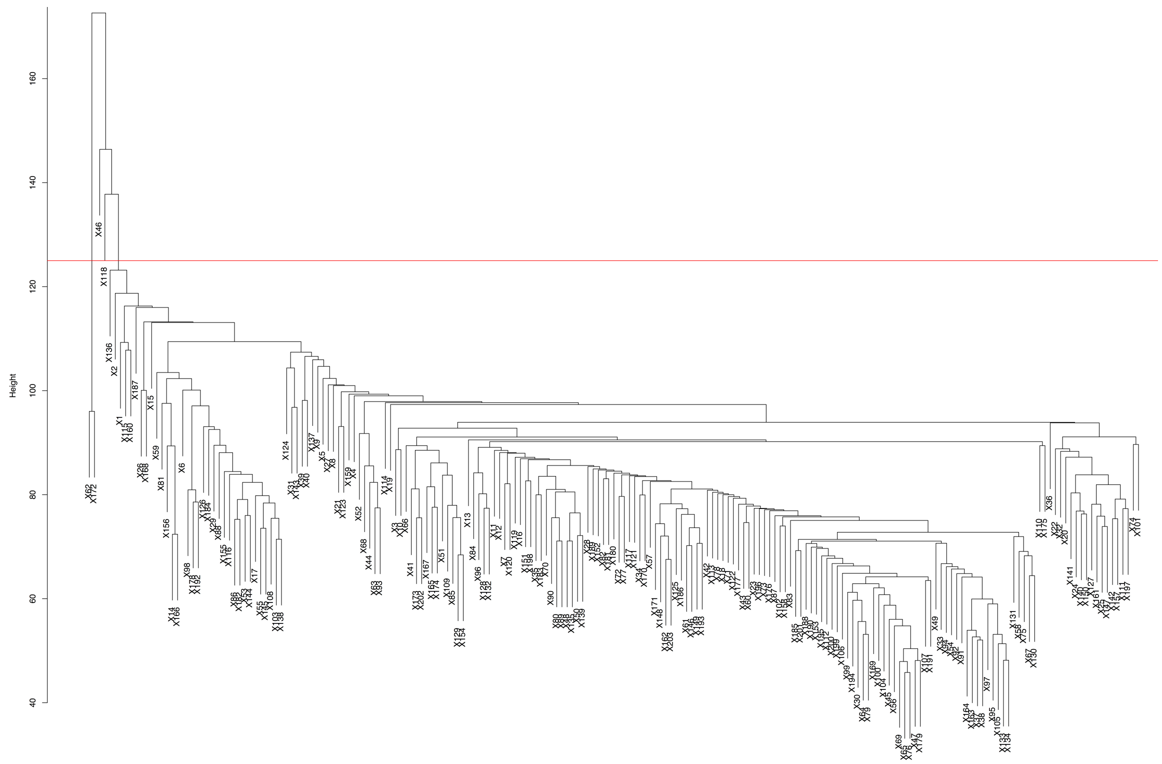

Figure 1. Hierarchical cluster dendrogram for 203 samples
Each branch represents a sample. The red horizontal line shows the cut height at which the sample tree was pruned.

To choose an appropriate soft-thresholding power, β, we used the pickSoftThreshold function to determine the β value that results in a network topology that fits well with a scale-free network, a network in which degree of connectivity follows a power function and results in hubs (genes with high degree of connectivity) and modules (network of highly connected genes). Based on a regression fitting index, we selected a β of 6 since this resulted in an R^2 = 0.854 and a mean connectivity of 25 (**Figure 2**).

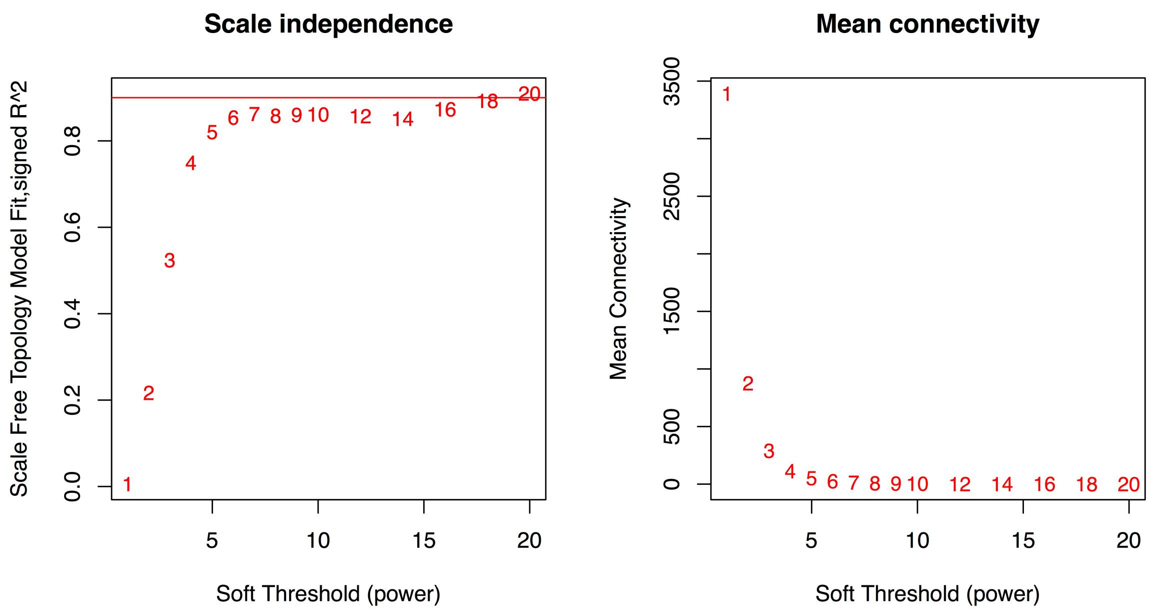

Figure 2. Soft-thresholding power selection
The panel on the left shows a plot between a range of candidate β values (from 1 to 20) and fit index (R^2) for a scale-free network property. The red line shows the R^2 = 0.9. The panel on the right plots the mean connectivities (average number of connection for each gene or probe in the network) as a function of β.

A pair-wise similarity matrix was computed using Pearson correlations. This was then transform into a 20,595 x 20,595 adjacency matrix by raising the similarity matrix to the power β = 6. The inter-connectedness (or topological overlap) between the CpGs was then calculated and hierarchical clustering was done to generate a gene tree or dendrogram using the flashClust function (**Figure 3**). Modules were the defined by dynamic tree cutting using the cutreeDynamic function. The minimum module size was set at 35 members and merging of modules was done at a minimum height of 0.2.

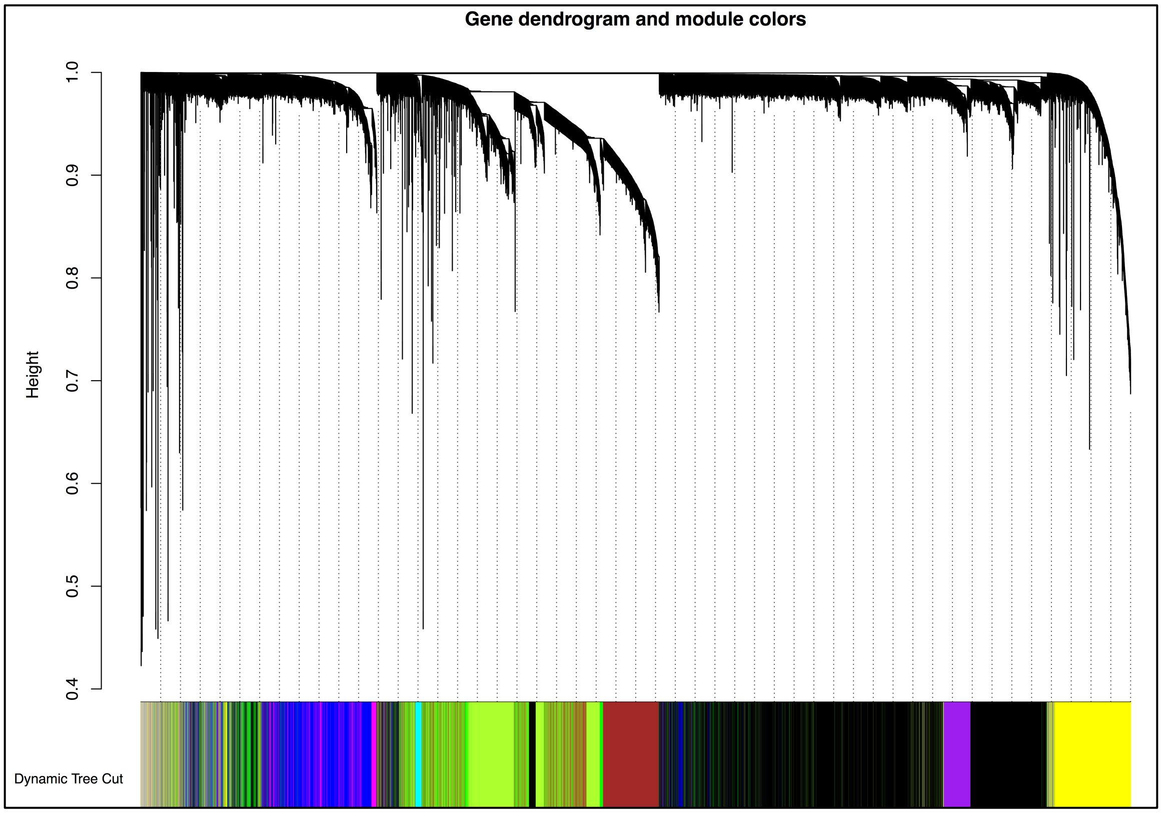

Figure 3. Dendrogram of CpG probes

Each branch in the dendrogram represents the clustering of correlated CpG sites. Gene modules defined from this are assigned colors and are shown at the bottom of the graph.
